# Social Cognition, Social Skill, and Social Motivation Minimally Predict Social Interaction Outcomes for Autistic and Non-Autistic Adults

**DOI:** 10.3389/fpsyg.2020.591100

**Published:** 2020-11-25

**Authors:** Kerrianne E. Morrison, Kilee M. DeBrabander, Desiree R. Jones, Robert A. Ackerman, Noah J. Sasson

**Affiliations:** School of Behavioral and Brain Sciences, The University of Texas at Dallas, Richardson, TX, United States

**Keywords:** social interaction, social cognition, social skills, first impressions, double empathy

## Abstract

Social cognition, social skill, and social motivation have been extensively researched and characterized as atypical in autistic people, with the assumption that each mechanistically contributes to the broader social interaction difficulties that diagnostically define the condition. Despite this assumption, research has not directly assessed whether or how these three social domains contribute to actual real-world social interaction outcomes for autistic people. The current study administered standardized measures of social cognition, social skill, and social motivation to 67 autistic and 58 non-autistic (NA) adults and assessed whether performance on these measures, both individually and relationally between dyadic partners, predicted outcomes for autistic and NA adults interacting with unfamiliar autistic and NA partners in a 5 minute unstructured “get to know you” conversation. Consistent with previous research, autistic adults scored lower than NA adults on the three social domains and were evaluated less favorably by their conversation partners. However, links between autistic adults' performance on the three social domains and their social interaction outcomes were minimal and, contrary to prediction, only the social abilities of NA adults predicted some interaction outcomes within mixed diagnostic dyads. Collectively, results suggest that reduced performance by autistic adults on standardized measures of social cognition, social skill, and social motivation do not correspond in clear and predictable ways with their real-world social interaction outcomes. They also highlight the need for the development and validation of more ecological assessments of autistic social abilities and the consideration of relational dynamics, not just individual characteristics, when assessing social disability in autism.

## Introduction

Autism Spectrum Disorder (ASD) is clinically defined in part by “persistent deficits in social communication and social interaction” (APA, 2013). Although the focus on communication and interaction necessitates consideration of interpersonal and relational dynamics, the focus on deficits—or reductions in normative characteristics presumed to underlie autistic disability—has historically centered research and treatment at the level of the individual. Indeed, a deep literature has accumulated cataloging how autistic people differ from non-autistic (NA) people in their neurology, cognition, and behavior (Pelphrey et al., 2004; Chevallier et al., 2012a; Morrison et al., 2017, 2019b), and a wide variety of intervention programs have been developed using this evidence base to try to normalize individual characteristics with the presumption that doing so may reduce or mitigate autistic disability (for a review, see Pallathra et al., 2019). For autistic adults without intellectual disability, most of these programs are psychosocial in nature and target three primary domains of social ability: social cognition, social skill, and social motivation. Each has been characterized as a core autistic deficit and are assumed to underlie the occupational challenges (Taylor et al., 2015), social isolation (Mazurek, 2014), and reduced quality of life (Billstedt et al., 2005) often experienced by autistic adults.

Social cognition refers to the perception and interpretation of social information (Brothers, 1990) and is often conceptualized as encompassing social perception (i.e., the prioritization and detection of social information), emotion recognition (i.e., accurately identifying the emotional state of others), and theory of mind (i.e., inferring the thoughts and intentions of other people; Baron-Cohen, 1991; Happé, 1994; Mathersul et al., 2013). On average, autistic adults score lower than NA controls on standalone assessments on each subdomain (Morrison et al., 2019b). They score lower on tasks assessing face recognition (e.g., Klin et al., 1999; Joseph and Tanaka, 2003), the identification of emotion from facial expressions, voices, and social scenes (e.g., Golan et al., 2007; Kennedy and Adolphs, 2012; Uljarevic and Hamilton, 2013; Sasson et al., 2016), and the inference of other peoples' intentions and mental states (e.g., Spek et al., 2010; Mathersul et al., 2013). Although these difficulties are presumed to mechanistically relate to the poor social and functional outcomes autistic adults often experience (Sasson et al., 2011), the surprisingly small number of studies that have empirically tested this assumption tend to find only modest relationships (Klin et al., 2002; Lerner and Mikami, 2012; Bishop-Fitzpatrick et al., 2014; Hanley et al., 2014; Deschrijver et al., 2016; Sasson et al., 2020), and no studies to our knowledge have tested whether individual social cognitive performance demonstrates meaningful associations to real-world social interaction for autistic adults. Given that social cognition is often targeted for improvement in psychosocial interventions as a means for enhancing social interaction, the lack of evidence in this regard reflects a significant oversight.

Social skill, meanwhile, is a broad umbrella term referring to the repertoire of behaviors used to navigate social demands and achieve social goals across varying contexts (Mueser and Bellack, 1998). A diverse set of skills have been conceptualized to comprise social skill, ranging from the use of interpersonal eye gaze to more complex competencies like negotiation ability (Mueser and Bellack, 1998; Nangle et al., 2010). Social skills reliably differ in autism (Constantino et al., 2000; Hus and Lord, 2014), with autistic adults often exhibiting non-normative social behaviors within social interactions relative to NA controls (Bishop, 1998; Patterson et al., 2001; Verhoeven et al., 2013). These differences can include atypical use of gaze, less observable conversational involvement, reduced verbal fluency, atypical affect, and asking fewer questions of their interaction partner (Ratto et al., 2011; Morrison et al., 2017). Training programs targeting social skills to improve social functioning among autistic adolescents and adults have yielded some limited benefits (Wykes et al., 2008; Bishop-Fitzpatrick et al., 2014), but they tend to lack generalizability to real-world outcomes (Palmen et al., 2010; Gates et al., 2017; Bottema-Beutel et al., 2018). This may stem in part from an overreliance on examining autistic social skill as an isolated, individual characteristic rather than assessing how it manifests within the context of actual interaction in which relational dynamics and not just individual behavior dictate outcomes (Milton, 2012; Bolis et al., 2018). It also suggests that a single, normative standard for social skill may not conform equally to the communication preferences and expectations of all groups or individuals. Indeed, this criticism is central to the Double Empathy theory of autism (Milton, 2012), which argues that social barriers between autistic and NA people are not solely driven by autistic misunderstanding of NA communication and behavior (as commonly described within autism research) but also the reverse: NA people often exhibit difficulty inferring the mental states and interpreting the social cues of autistic people (Edey et al., 2016; Alkhaldi et al., 2019). From this perspective, social skill is relative, contextual, and necessitates a focus on relational dynamics rather than individual ability.

Finally, social motivation refers to the seeking and liking of social information and relationships (Berridge et al., 2009; Chevallier et al., 2012a). Young children on the autism spectrum often demonstrate reduced attention and divergent reward responses for social information from early in life (Baranek, 1999; Pierce et al., 2011; Chevallier et al., 2012a; Moriuchi et al., 2017), which is theorized to produce cascading effects on developing social neural networks that manifest over time in divergent social behaviors and social cognitive abilities relative to same age peers (Dawson et al., 2005; Chevallier et al., 2012a). In older autistic children and adolescents, some work suggests that diminished social motivation may result in fewer social exchanges and less effort toward maintaining relationships (e.g., Chevallier et al., 2012b). However, many other studies have found that social motivation is highly variable among autistic adolescents and adults (Garman et al., 2016), most of whom express similar desires for friendships and relationships as their NA peers (Bauminger and Kasari, 2000; Whitehouse et al., 2009; Lasgaard et al., 2010; Mazurek, 2014). Higher social motivation among autistic individuals may relate to having better quality friendships, engaging in more social interactions, and displaying higher rates of prosocial behavior in interactions with others (Chevallier et al., 2012b; Dean et al., 2014; Sedgewick et al., 2016). At the same time, lower or different social interest is not inherently negative (Dawson and Cowen, 2019; Fletcher-Watson and Crompton, 2019), and pressure to conform to normative expectations can be detrimental to autistic well-being (Cage and Troxell-Whitman, 2019). For instance, many autistic adolescents and adults without intellectual disability report engaging in effortful and often exhausting “camouflaging” behaviors in order to appear more typical within social interactions (Hull et al., 2017). These deliberate masking behaviors suggest that—rather than lacking motivation for relationships—autistic individuals may instead differ in their social skill and communication styles, struggle to have their social needs met, and expend tremendous effort trying to fit in (Hintzen et al., 2010; Chevallier et al., 2012b; Garman et al., 2016).

Collectively, this broad body of research has delineated reliable group-level differences between autistic and NA people on a range of social cognitive, social skill, and social motivation measures, with findings more variable and idiosyncratic concerning their association with social and functional outcomes. One potential reason for the lack of more established links with broader outcomes is that no studies have assessed whether and how these three social abilities relate to social interaction success or difficulties for autistic adults. Social interaction serves as the interface between the individual abilities and social outcomes, and despite social interaction difficulties constituting a core diagnostic component of autism, no research to our knowledge has systematically examined whether social cognition, social skill, and social motivation correspond to real-world social interaction outcomes for autistic adults. Critically, social interaction involves more than one person and necessitates consideration of not just an individual's social abilities, but also those of the interaction partner—and the relational combination between them—in order to understand how each partner influences the other (De Jaegher, 2013; Hehman et al., 2017). Research in autism, however, has focused overwhelmingly at the level of the individual, with studies of social interaction even being called a “blind spot” (De Jaegher, 2013) because so few studies have assessed dynamic interaction amongst and between autistic people.

This has started to change in recent years. Recent empirical work with autistic adults has shown that social interaction quality and positive perceptions are driven by relational factors to a greater degree than individual ones (Crompton et al., 2020; Morrison et al., 2020). For instance, autistic adults disclose more about themselves (Morrison et al., 2020), communicate more effectively, and establish better rapport (Crompton et al., 2020) when interacting with other autistic adults relative to NA adults. This suggests that relational compatibility, and not just individual characteristics, contribute to social interaction outcomes for autistic adults, but it remains unclear whether specific social abilities either individually or dyadically underlie this effect.

A previous study (Morrison et al., 2020) reported that autistic adults were evaluated less favorably by both autistic and NA partners after engaging in a real-world “get to know you” conversation, and NA adults expressed a preference for future social interaction with NA relative to autistic adults. In contrast, autistic adults trended toward preferring interaction with autistic relative to NA adults. The current study analyzes additional data from this sample to assess whether and how three aspects of social ability (i.e., social cognition, social skills, and social motivation) relate to social interaction outcomes for autistic and non-autistic adults across three dyadic combinations of diagnostic status (i.e., A-A, NA-NA, A-NA). The Actor-Partner Interdependence Model (APIM; Kenny et al., 2006) was used to assess the effect of individuals' social abilities on their own evaluations of their partner and the interaction (actor effects), the effect of the partners' social abilities on how individuals evaluate that partner and the interaction (partner effects), and the interaction between the two (actor-partner interactions). We predicted that (1) regardless of diagnosis, individuals with lower social cognitive performance, social motivation, and observed social skill will evaluate their partner less favorably and rate their own experience of the interactions lower in quality and closeness (i.e., actor effects); (2) regardless of diagnosis, individuals with lower social cognitive performance, social motivation, and observed social skill will be evaluated less favorably by their partners and their partners would rate their experience of the interactions lower in quality and closeness (i.e., partner effects); (3) actor, partner, and actor-partner interaction effects involving social variables will be moderated by diagnosis, such that effects of social abilities on outcomes will be stronger for autistic compared to NA individuals; and (4) actor, partner, and actor-partner interaction effects involving social variables will be moderated by dyad type, such that the effect of social abilities on outcomes will differ depending on whether dyads share or differ in their diagnostic status. Together, these hypotheses assess which factors predict person and interaction evaluations, and what combinations of partners and/or traits lead to poor or favorable interactions.

## Methods

### Participants

One hundred and twenty-five adults (67 A, 58 NA) participated in one of three types of conversation dyads: A-A (*n* = 22), A-NA (*n* = 25), and NA-NA (*n* = 23). Autistic participants were recruited from the UT Dallas Autism Research Collaborative, a research registry of clinically assessed autistic adults who have expressed interest in participating in university research studies. Inclusion in the registry requires confirmed diagnoses using the ADOS-II (Lord et al., 2000) and full-scale intelligent quotients (IQ) over 70 on the WASI-II (Wechsler, 2011), both of which occurred during a previous clinical intake session. Full-scale IQs over 90 were required for this study in order to be intellectually comparable to the NA participants. All included NA participants were university undergraduates, and only those who reported no history of psychiatric illness (8 excluded), no developmental disability (including autism; 1 excluded), and no autistic first-degree relatives (4 excluded) were retained in the study. Those with autistic first-degree relatives were excluded to minimize inclusion of NA adults with high familiarity with autism and/or autistic traits. Additionally, two autistic adults were excluded for having an IQ lower than 70. The protocol for the study was approved by the University Institutional Review Board, and all participants provided informed consent before the study began.

Autistic and NA participants were recruited with the intent of matching the diagnostic groups and the three dyad types demographically. All participants were male to avoid the complicating dynamics of inter-sex dyads and because the higher male ratio in autism (Fombonne, 2009) and in our recruitment sources precluded a well-powered examination of gender effects. Autistic and NA participants differed in age (A mean = 23.51, SD = 4.07; NA mean = 20.84, SD = 3.17; *p* < 0.01) but did not differ on race (A = 84% White; NA = 81% White; *p* = 0.83) and estimated IQ on the Wide Range Achievement Test (WRAT-3; Wilkinson, 1993; A mean = 110.77, SD = 8.58; NA mean = 109.91, SD = 8.39; *p* = 0.58), a brief assessment that correlates highly with full scale IQ (Powell et al., 2002). The three dyad types did not differ on race (*p* = 0.97) or estimated IQ (*p* = 0.17), but did on age (*p* < 0.01), with the NA-NA group consisting of younger participants than the other two dyad types. To ensure that any findings between diagnostic groups and dyad types were not influenced by demographic characteristics, age, race, and IQ were covaried in all analyses. Demographic characteristics for the diagnostic and dyad groups can be viewed in Table 1. For more details about the sample, including information about the descriptive and psychometric properties of all included measures, see Morrison et al. (2020).

**Table 1:** Demographic characteristics for diagnostic and dyad groups.

	**Dyad groups**								**Overall**			
	**A-A**		**NA-NA**		**A-NA**				**A**		**NA**	
	**(*****n*** **= 42)**		**(*****n*** **= 40)**		**(*****n*** **= 42)**				**(*****n*** **= 66)**		**(*****n*** **= 58)**	
**Race**												
White	36		33		34				56		47	
Black	2		2		2				3		3	
Asian	2		1		2				3		2	
Other	2		4		4				4		6	
					**A**		**NA**					
	***M***	***SD***	***M***	***SD***	***M***	***SD***	***M***	***SD***	***M***	***SD***	***M***	***SD***
Age	22.67	3.62	20.62	3.43	25.10	4.47	21.33	2.50	23.51	4.07	20.84	3.17
WRAT-3 IQ	111.88	7.12	110.78	7.91	108.67	10.72	108.00	9.34	110.77	8.58	109.91	8.39

*WRAT-3, Wide Range Achievement Test 3rd Edition*.

#### Procedure

Potential participants were initially screened for inclusion criteria, scheduling availability, and demographic characteristics. This information was then used to recruit unfamiliar dyadic partnerships of participants similar on age and race. Efforts to recruit unfamiliar conversation partners were largely successful: only one dyad consisted of partners who mutually acknowledged seeing their conversation partner previously, but both said that they had never spoken.

After providing informed consent, participants sat in chairs facing each other and were videotaped while completing an unstructured conversation developed to evaluate dyadic interaction (Berry and Hansen, 1996). This measure originally was created to measure interactions between two NA participants but recently similar paradigms have also been used with autistic adults (Usher et al., 2018; Morrison et al., 2020). Participants are told that they will be talking to their partner for 5 min. No specific instructions are given other than telling them that their goal is to get to know the other person. After instructions were given, the experimenter moved behind a partition to avoid influencing the interaction. Participants were not explicitly made aware of the diagnostic status of their partners, but disclosure occurred organically during interactions within three A-A dyads and three A-NA dyads. Following the conversation, participants completed measures on separate computer stations that (1) recorded their impressions of the interaction and their conversation partner, (2) assessed their social cognitive performance, and (3) measured their social motivation. To ensure order effects did not influence results, these groups of measures were counterbalanced for each participant, and the order of the measures within each group of measures was randomized.

### Measures

#### Evaluation of the Partner and the Interaction

Participants evaluated their conversation partner using the First *Impression Scale for Autism* (FIS; Sasson et al., 2017) and *the International Personality Item Pool—Interpersonal Circumplex (IPIP-IPC;* Markey and Markey, 2007) The FIS includes 10 items using a four-point scale. Participants rated their partner on six traits (awkwardness, attractiveness, trustworthiness, likability, dominance, and intelligence), and completed four items concerning their interest in socializing with their partner in the future (e.g., “I would hang out with this person in my free time”). Because the social interest items, but not the trait items, showed relatively high internal consistency (see Morrison et al., 2020 for details), a composite score averaging the four social interest items was used in analyses, whereas the six trait items were individually included.

The IPIP-IPC consists of 32 items assessing social behavioral characteristics unassessed by the FIS. Specifically, the IPIP-IPC measures interpersonal warmth and dominance, two key predictors of dyadic behavior in social interactions and a variety of interaction outcomes [e.g., relationship satisfaction, task productivity, and liking (Markey and Markey, 2007; Markey et al., 2010)]. Items are aggregated to calculate separate warmth and dominance scores that are then used in analyses.

Participants evaluated qualities of the interaction using *the Social Interaction Evaluation Measure* (SIEM: Berry and Hansen, 1996), the *Inclusion of Other in the Self* (IOS Scale; Aron et al., 1997), and the *Subjective Closeness Index* (SCI; Berscheid et al., 1989). The SIEM is a self-report measure consisting of 11 questions rated on an eight-point scale concerning the participant's perceptions of the interaction quality, the intimacy of the interaction, the partner's level of disclosure, and the partner's level of engagement in the conversation. The items are averaged to create a composite score indicating overall interaction quality (Heerey and Kring, 2007).

The IOS and SCI are measures of interpersonal closeness that are averaged together to create an overall closeness composite score (Aron et al., 1997). The IOS requires the participant to select one of seven overlapping circles that best represents how close they feel to their conversation partner. The SCI uses a seven-point scale to ask the participant to rate their level of closeness to their partner relative to their other relationships and their perception of closeness in the relationships of other people.

#### Evaluation of Social Abilities: Social Cognition, Social Motivation, and Social Skills

##### Social cognition

Participants completed three measures spanning the separate domains of social cognition: face perception (Benton Facial Recognition Task; Benton et al., 1983), emotion recognition (Penn Emotion Recognition Task, ER-40; Kohler et al., 2000), and theory of mind (The Awareness of Social Inference Task. TASIT; McDonald et al., 2003). In the Benton, participants view 54 faces and select the matching face from an array of six faces. In the ER-40, participants select one of five emotion choices corresponding to the emotion expressed in 40 face photos. In the TASIT, participants watch 16 short videos depicting characters lying or being sarcastic within social interactions and answer four questions after each video regarding what the characters' intentions, thoughts, and beliefs were about the other people or the scenario. All three tasks have been used in previous studies of autism (Philip et al., 2010; Neves et al., 2011; Ratto et al., 2011) and have been psychometrically validated for inclusion in autism research (Morrison et al., 2019b). As has been done previously (Sasson et al., 2013), social cognitive scores from these three domains were standardized and averaged together to yield a total social cognition composite score used in primary analyses. The independent impact of each social cognition domain on social interaction outcomes was also pursued in exploratory analyses.

##### Social motivation

Participants completed the Friendship Motivation Scale (Richard and Schneider, 2005) to assess their interest in forming social relationships. Participants answer 12 questions on a 4-point scale across four subscales: intrinsic motivation, identified regulation, external regulation, and amotivation. Intrinsic motivation refers to self-determination for seeking friendships, in which social relationships are satisfying for internal reasons (e.g., for the pleasure I get by talking with friends). The other three types of motivation are extrinsic in nature. Identified regulation refers to seeking relationships for their own sake (e.g., because I think having friends is good for me). External regulation refers to seeking friendships for environmental reasons or rewards (e.g., to be invited to parties). Lastly, amotivation refers to a lack of motivation to seek friendships because the individual does not perceive benefits from friendships (e.g., I don't see why I would want to have friends). The total social motivation score was computed by summing weighted subscale scores, with higher scores signifying higher social motivation (see Richard and Schneider, 2005 for formula).

##### Social skills

To obtain a measure of both partners' social skills, three independent raters (one autistic) were trained on the Conversation Probe (CP) social behavior coding manual (Pinkham and Penn, 2006). Prior to coding, raters attended training sessions and coded videos until consensus in ratings was achieved on 20% of the videos. All raters were blind to participant diagnoses. The CP captures both discrete social skill ratings and a holistic rating of the participant's overall social skill. Coders first coded nine discrete behaviors categorized into four composite skill groups: appropriate content, paralinguistic behaviors, interactive behaviors, and non-verbal behaviors (Morrison et al., 2017). Conversational content refers to the participant's ability to discuss topics appropriate to meeting someone for the first time. Paralinguistic behaviors quantify the quality of participants' speech other than semantic content (e.g., speaking with clarity, enunciating clearly and fluently, and successfully switching turns with their partner). Interactive behaviors measure the degree to which participants are interested in getting to know their partners and carry on the interaction. This subscale was comprised of involvement, or the degree to which the participants appear engaged in the conversation, and the number of questions the participants asked of their partner. Lastly, non-verbal behaviors consisted of the degree of appropriate eye-contact and affective behaviors displayed by the participants. These social behaviors were originally derived based upon non-autistic norms, and thus the CP should be understood as measuring social skills considered normative and valued by non-autistic society.

Each social skill rating was made on a nine-point Likert scale, where higher scores indicated better social skills ability. Coders also make a holistic judgement of the participant's overall skill ability, rating how successful the participant was at interacting with his partner. Intraclass correlation coefficients (ICCs) were computed to assess reliability on the videos. The three coders' consistency ranged from 0.57 to 0.95 on the behaviors across the full sample of videos and they were strongly consistent for overall social skills (ICC = 0.732). Reliability is displayed in Table 2.

**Table 2:** Means and group comparison of social skills.

	**ICC**	**A**		**NA**		***F*_**(1, 123)**_**	***p***
		***M***	***SD***	***M***	***SD***		
Content	0.731	6.72	0.81	7.09	0.60	8.177	0.005
Clarity	0.588	6.08	0.96	6.42	0.72	4.934	0.028
Fluency	0.765	6.01	1.08	6.60	0.59	13.817	<0.001
Meshing	0.713	6.02	1.16	6.59	0.68	10.841	0.001
Gaze	0.660	6.67	1.12	7.55	0.57	29.398	<0.001
Involvement	0.793	6.59	1.09	7.21	0.55	15.149	<0.001
Asks questions	0.951	3.76	2.66	5.61	2.42	16.297	<0.001
Appropriate affect	0.655	6.80	0.54	7.12	0.46	12.361	0.001
Flat affect	0.712	5.85	1.00	6.27	0.82	6.548	0.012
Social anxiety	0.725	6.01	0.97	6.78	0.64	27.012	<0.001
Overall skill	0.732	5.57	1.04	6.44	0.62	31.494	<0.001
Repetitive verbal content	0.566	6.79	0.77	7.18	0.42	11.688	0.001
Repetitive movement	0.743	6.56	0.97	7.17	0.55	17.813	<0.001
Verbosity	0.905	6.36	1.85	6.40	1.13	0.022	0.882
Paralinguistic	–	6.04	0.86	6.54	0.47	15.675	<0.001
Non-verbal	–	6.44	0.69	6.98	0.47	25.459	<0.001
Interactive	–	5.18	1.60	6.41	1.34	21.373	<0.001

*ICC refers to Intraclass correlation coefficient for coders' reliability. Note the paralinguistic, non-verbal, and interactive behaviors are composite scores rather than coded items, and thus do not have ICCs*.

### Analysis Plan

Before proceeding to our primary analyses, we inspected the descriptive statistics for the study variables and tested whether autistic and NA adults significantly differed in their respective group means. We then investigated the pattern of zero-order correlations between the study variables for autistic and NA adults separately to gain some preliminary insights into possible group differences in the predictor-predictor and predictor-outcome associations.

**Figure 1:**
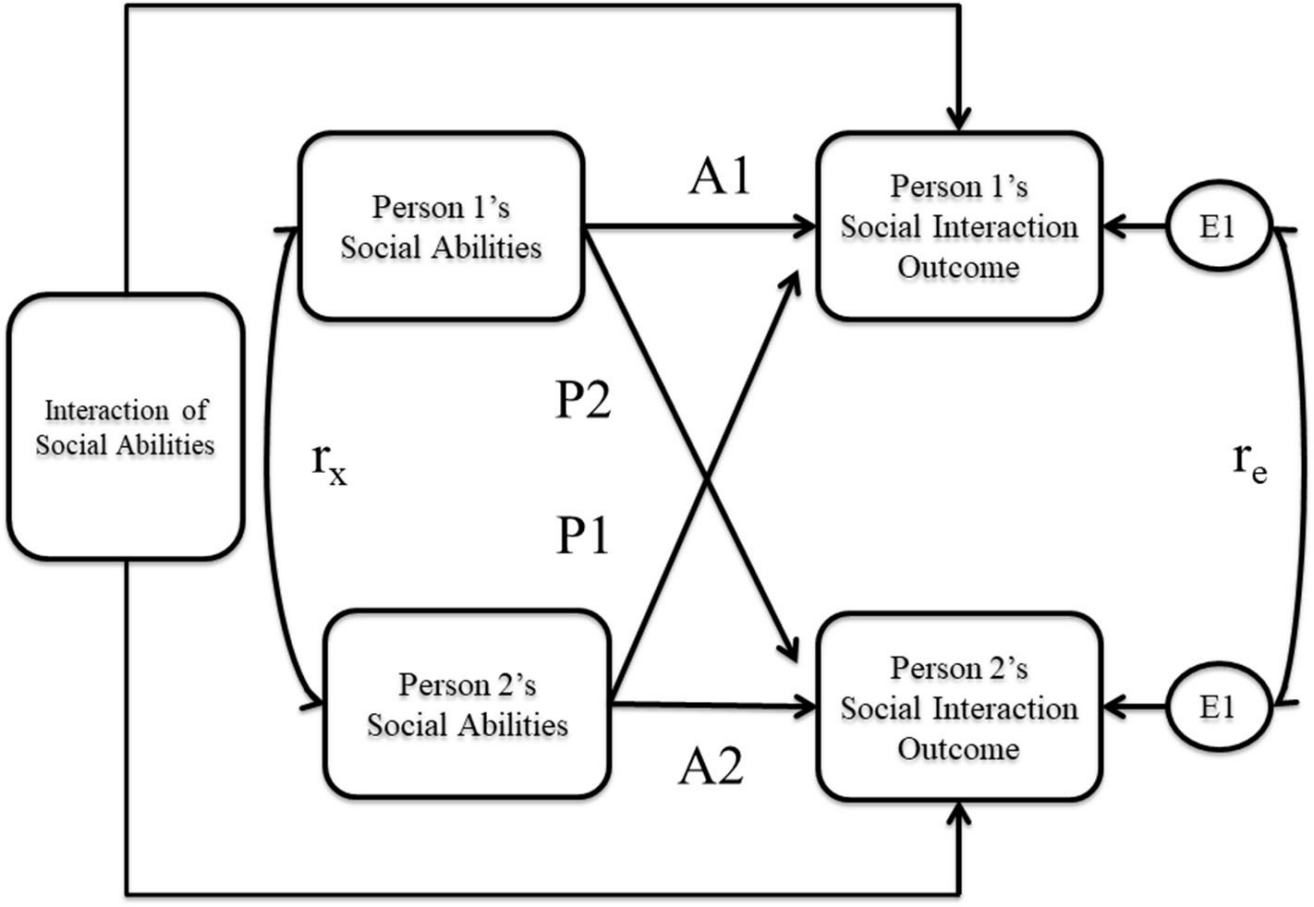
Actor partner interdependence model (APIM) predicting social interaction outcomes with individual and partner social abilities. A-paths represent the actor effects and P-paths represent the partner effects. The interaction term represents the effect of the individual's social abilities on the individual's social interaction outcome depending on the partner's social abilities.

Because outcomes for partners were interrelated and thus non-independent, traditional analytic techniques (e.g., general linear model) could not be used for primary analyses. Instead, the Actor-Partner Interdependence Model (APIM) for indistinguishable dyads was used (Kenny et al., 2006). The APIM provides estimates of actor effects (e.g., the effect of individuals' social abilities on their own interaction outcomes), partner effects (e.g., the effect of individuals' partners' social abilities on individuals' interaction outcomes), and (if researchers are interested) actor-partner interactions (e.g., how the effect of individuals' social abilities on their own interaction outcomes depends upon their partners' social abilities). Additionally, by collecting dyads that differed in their diagnostic composition, we could investigate whether effects differed for autistic adults compared to NA adults, as well as whether the particular combination of dyad members (i.e., A-A, A-NA, NA-NA) moderated any effects (Kraemer and Jacklin, 1979; Kenny et al., 1988). Figure 1 visually displays the model used for analysis.

APIMs were specified using multilevel modeling with Restricted Maximum Likelihood estimation in SPSS Version 25. Multilevel modeling is appropriate because participants and their interaction partners are nested within dyads. It also helps to account for missing data in the outcomes, which were minimal in this study. Actor IQ, race, and age were entered as co-variates in all analyses. To facilitate the interpretation of the coefficients in each of the APIM analyses, continuous predictors were grand-mean centered and categorical predictors were effects coded (moreover, interaction terms assessing moderation were specified using these centered and effect-coded variables). An adjusted alpha of 0.01 was used as the threshold for statistical significance given the large number of tests; however, a more lenient alpha of 0.05 was used when significant interaction terms were followed up to increase our power to detect simple slopes once our more conservative threshold for detecting an interaction was reached.

## Results

### Descriptive Statistics

Normality, skew, and kurtosis were within acceptable ranges for analyses. Skew and kurtosis values were below the absolute value of 2 for all measures, with two item-level exceptions: aggression/dominance and the behavioral intent composite from the first impression scale exceeded the kurtosis threshold but were still relatively normal in their distributions. Means and standard deviations for social cognitive tasks, social motivation, and social skills can be viewed in Tables 2, 3 [those for outcome measures (i.e., first impression scale, IPC warmth, IPC dominance, interaction quality, and closeness) appear in Morrison et al., 2020]. NA adults scored higher than autistic adults on the Benton [*F*_(1, 177)_ = 26.37, *p* < 0.001], TASIT [*F*_(1, 117)_ = 14.98, *p* < 0.001], FMS [*F*_(1, 117)_ = 12.46, *p* = 0.001], social cognition composite score [*F*_(1, 117)_ = 26.02, *p* < 0.001], overall social skills ratings [*F*_(1, 123)_ = 31.49, *p* < 0.001], but diagnostic groups did not differ on the ER-40 [*F*_(1, 117)_ = 2.79, *p* = 0.10].

**Table 3:** Scores on predictors for diagnostic and dyad groups.

	**A-A dyads**		**NA-NA dyads**		**A-NA dyads (A left; NA right)**				**A overall**		**NA overall**	
	**(*****n*** **= 42)**		**(*****n*** **= 40)**		**(*****n*** **= 42)**				**(*****n*** **= 66)**		**(*****n*** **= 58)**	
	***M***	***SD***	***M***	***SD***	***M***	***SD***	***M***	***SD***	***M***	***SD***	***M***	***SD***
Benton	43.35	4.26	47.35	3.64	43.14	3.95	46.00	3.43	43.28	4.12	46.93	3.60
TASIT	51.60	6.54	56.33	4.39	53.14	6.38	55.22	3.06	52.13	6.48	55.98	4.03
ER-40	34.08	2.49	34.73	2.72	33.52	2.73	34.56	2.28	33.89	2.57	34.67	2.57
Social Cog	−0.24	0.66	0.40	0.60	−0.24	0.70	0.21	0.47	−0.24	0.66	0.34	0.57
FMS	15.35	7.15	19.40	5.29	16.33	7.16	20.33	4.45	15.69	7.11	19.69	5.02
Overall_SS	5.71	1.00	6.35	0.63	5.67	0.96	6.65	0.56	5.57	1.04	6.44	0.62

*M, Mean; SD, Standard Deviation; ER-40, Emotion Recognition test; FMS, Friendship Motivation Scale; SS, Social Skills; TASIT, The Awareness of Social Inference Test*.

Social ability predictors were weakly to moderately correlated with one another (Table 4). Correlations between predictors and actor and partner outcomes showed that for autistic adults, several social abilities moderately predicted partner evaluations (Tables 5, 6). As can be seen in Table 5, for autistic adults, higher social motivation on the FMS was related to perceiving the partner as warmer, less aggressive/dominant, smarter, and having a stronger desire to have a conversation with their partner. ER-40 scores predicted stronger acceptance of living near the partner, but higher theory of mind performance on the TASIT was related to feeling less close to partners, perceiving the partner as less dominant, and having less desire hang out with their partners later. For NA adults, higher ER-40 scores predicted rating partners lower in warmth, and higher TASIT scores were related to perceiving the partner as less dominant and more trustworthy. Higher social motivation on the FMS was related to seeing the partner as more attractive. As can be seen in Table 6, autistic adults with better observed social skills were rated as less awkward, smarter, and having higher quality interactions, those with better theory of mind performance on the TASIT were rated as smarter, and those with higher emotion recognition scores on the ER-40 were rated less dominant and more awkward. For NA adults, those with higher Benton facial recognition scores were rated as warmer.

**Table 4:** Correlations between predictors.

	**Benton**	**TASIT**	**ER-40**	**FMS**	**Overall SS**
Benton	1	0.017	0.361^**^	−0.193	0.035
TASIT	0.272^*^	1	0.221	−0.24	0.122
ER-40	0.121	0.308^*^	1	−0.009	0.196
FMS	0.129	0.166	0.278^*^	1	−0.037
Overall SS	0.06	0.335^**^	−0.137	−0.025	1

NA correlations are above diagonal and A below it. Predictors are actor social abilities. FMS, Friendship Motivation Scale; ER-40, Emotion Recognition task; SS, Social Skill; TASIT, The Awareness of Social Inference test.

* p < 0.05,

** *p < 0.01*.

**Table 5:** Correlations between actor social abilities with actor outcomes.

**NA**	**Benton**	**TASIT**	**ER-40**	**FMS**	**Overall social skill**
Interaction quality	−0.068	0.058	−0.014	0.141	−0.048
Closeness	0.09	−0.152	−0.185	0.038	−0.174
IPC warmth	−0.159	0.096	−0.299^*^	0.004	−0.106
IPC dominance	−0.141	−0.313^*^	−0.181	−0.038	0.084
Awkward_R	0.039	−0.085	0.041	−0.034	−0.064
Attractive	−0.108	0.177	0.118	0.263^*^	0.062
Trustworthy	−0.214	0.300^*^	−0.147	−0.077	0.008
Aggressive/Dominant	−0.18	−0.188	0.053	0.008	−0.245
Likable	−0.087	0.099	−0.116	0.178	−0.001
Smart	−0.031	0.232	0.027	0.118	0.211
Live near	−0.108	0.235	−0.133	0.078	0.046
Hangout	0.208	0.178	−0.064	0.153	−0.039
Sit near	−0.05	0.138	−0.018	0.177	−0.094
Conversation	0.256	0.202	0.071	0.166	0.232
Behavioral Intent	0.094	0.278^*^	−0.062	0.209	0.041
**A**					
Interaction quality	0.143	0.013	−0.03	0.089	−0.135
Closeness	−0.093	−0.254^*^	−0.131	0.006	0.012
IPC warmth	0.019	−0.053	−0.166	0.409^**^	−0.021
IPC dominance	0.062	−0.316^*^	−0.153	−0.168	0.014
Awkward_R	−0.16	−0.105	−0.096	−0.026	−0.126
Attractive	0.044	0.024	0.018	−0.121	0.056
Trustworthy	−0.128	−0.051	−0.173	0.049	0.15
Aggressive/Dominant	0.042	0.132	0.042	−0.243^*^	0.22
Likable	−0.087	−0.190	−0.052	0.081	−0.006
Smart	−0.059	−0.015	0.135	0.290^*^	−0.008
Live near	0.087	0.002	0.250^*^	0.18	0.053
Hangout	−0.149	−0.305^*^	−0.038	0.19	−0.157
Sit near	0.111	−0.004	0.131	0.232	−0.049
Conversation	0.024	−0.162	−0.052	0.448^**^	0.037
Behavioral intent	0.039	−0.150	0.127	0.366^**^	−0.035

Outcomes are actor ratings of the partner and interaction. Awkward was reverse scored. ER-40, Emotion Recognition task; FMS, Friendship Motivation Scale; IPC, Interpersonal Circumplex; TASIT, The Awareness of Social Inference test.

* p < 0.05,

** *p < 0.01*.

**Table 6:** Correlations between actor predictors and partner outcomes.

**NA**	**Benton**	**TASIT**	**ER-40**	**FMS**	**Overall social skill**
Interaction quality	0.119	0.137	0.118	−0.053	0.123
Closeness	0.082	0.071	0.028	−0.042	0.109
IPC warmth	0.297^*^	0.132	0.193	0.109	0.256
IPC dominance	0.136	0.162	0.095	0.031	0.229
Awkward_R	0.206	0.137	0.106	−0.091	0.233
Attractive	−0.144	−0.114	0.067	0.225	0.115
Trustworthy	0.078	−0.178	0.220	0.274	−0.095
Aggressive/Dominant	−0.061	0.22	−0.013	0.099	−0.017
Likable	−0.120	0.105	0.027	0.041	0.096
Smart	−0.020	−0.03	0.057	0.24	−0.064
Live near	−0.053	−0.035	0.009	0.163	0.043
Hangout	0.079	0.145	−0.102	−0.051	0.129
Sit near	0.035	0.068	0.150	−0.071	−0.220
Conversation	0.204	−0.153	0.013	−0.09	−0.005
Behavioral Intent	0.073	0.026	0.025	0.001	−0.017
**A**					
Interaction quality	−0.013	−0.004	0.010	−0.063	0.260^*^
Closeness	0.188	0.144	0.192	0.054	0.073
IPC warmth	0.006	0.036	0.162	−0.086	0.221
IPC dominance	0.04	−0.185	−0.267^*^	−0.093	0.060
Awkward_R	0.041	0.094	−0.262^*^	−0.029	0.328^*^
Attractive	−0.107	0.101	−0.045	−0.2	0.224
Trustworthy	−0.022	−0.184	−0.147	0.095	0.206
Aggressive/Dominant	0.221	−0.067	−0.201	0.027	0.096
Likable	0.074	0.059	0.150	0.026	0.059
Smart	0.054	0.348^**^	0.192	0.149	0.265^*^
Live near	−0.029	0.063	0.142	0.168	−0.147
Hangout	0.22	0.22	0.023	0.058	0.149
Sit near	−0.081	0.033	0.070	0.189	−0.045
Conversation	0.041	−0.047	−0.071	0.135	0.227
Behavioral Intent	0.047	0.109	0.079	0.234	0.052

Outcomes are partner ratings of the actor and interaction. Awkward was reverse scored. ER-40, Emotion Recognition task; FMS, Friendship Motivation Scale; IPC, Interpersonal Circumplex; TASIT, The Awareness of Social Inference test.

* p < 0.05,

** *p <0.01*.

### Actor-Partner Interdependence Model (APIM) Analyses

An initial model, detailed in Morrison et al. (2020), was run to assess the effect of the diagnostic status (A or NA) of the actor, partner, and the interaction between them on reports of social interaction quality, closeness, and first impressions of various traits. For the current study, this model was run with additional parameters to examine (a) if social abilities (i.e., social cognition, social motivation, and social skill) predicted social interaction outcomes, and (b) if these effects are moderated by diagnosis and dyad type (see Tables 7–15). Each of the tables focuses on a different set of social ability predictors and social interaction outcome variables. Whereas, Tables 7–9 includes APIM analyses with social evaluation measures as outcome variables, Tables 10–15 include APIM analyses with first impression measures as outcome variables. Further, each table specifies the particular social ability variables being used as predictors (i.e., social cognitive, social skills, or social motivation).

**Table 7:** Actor-partner interdependence model analyses estimating the combinatorial effects of diagnostic status and social cognition variables on the social evaluation outcomes of closeness, interaction quality, warmth, and dominance.

	**Social evaluation outcomes**							
**Predictors**	**Closeness**		**Interaction quality**		**IPC warmth**		**IPC dominance**	
**Social-cognition predictors**	***b***	**SE**	***b***	**SE**	***b***	**SE**	***b***	**SE**
Intercept	2.92	0.23	5.70	0.18	0.03	0.17	−0.03	0.20
**Demographic variables**								
Actor WRAT	−0.02	0.01	−0.01	0.01	0.01	0.01	−0.01	0.01
Actor race—AfricanAmerican	−0.01	0.42	0.11	0.34	0.37	0.31	0.06	0.36
Actor race—Asian	0.18	0.46	−0.51	0.37	−0.35	0.34	−0.33	0.39
Actor race—Other	0.05	0.34	0.52	0.29	0.09	0.27	0.32	0.31
Actor age	0.05	0.03	0.06^*^	0.02	0.06^*^	0.02	0.02	0.03
**Diagnosis variables**								
Actor diagnosis	0.24	0.15	−0.03	0.13	−0.20	0.12	−0.16	0.14
Partner diagnosis	−0.04	0.14	−0.12	0.12	−0.14	0.11	−0.17	0.13
Actor^*^Partner diagnosis	0.19	0.16	0.12	0.13	0.03	0.12	0.03	0.14
**Social cognition variables**								
Actor SC	−0.13	0.26	0.43	0.22	0.06	0.21	−0.21	0.24
Partner SC	0.38	0.26	0.13	0.22	0.40	0.21	−0.15	0.24
Actor^*^Partner SC	−0.30	0.39	0.14	0.32	0.09	0.29	0.31	0.34
**Moderation of social cognition variables by diagnosis variables**								
Actor diagnosis^*^Actor SC	−0.12	0.26	−0.25	0.22	−0.10	0.20	−0.05	0.24
Actor diagnosis^*^Partner SC	0.06	0.27	−0.08	0.23	0.15	0.21	−0.14	0.25
Actor diagnosis^*^(Actor^*^Partner SC)	−0.02	0.32	−0.30	0.28	−0.03	0.26	0.09	0.31
Partner diagnosis^*^Actor SC	−0.04	0.28	0.43	0.23	0.18	0.22	0.18	0.26
Partner diagnosis^*^Partner SC	−0.03	0.26	−0.16	0.22	−0.31	0.20	−0.14	0.24
Partner diagnosis^*^(Actor^*^Partner SC)	−0.02	0.32	0.53	0.28	0.41	0.26	0.10	0.31
**Moderation of social cognition variables by dyad type**								
(Actor^*^Partner diagnosis)^*^Actor SC	−0.22	0.28	−0.69^**^	0.24	−0.34	0.22	−0.40	0.26
(Actor^*^Partner diagnosis)^*^ Partner SC	−0.15	0.28	−0.17	0.23	−0.27	0.22	0.05	0.25
(Actor^*^Partner diagnosis)^*^ (Actor^*^Partner SC)	−0.14	0.38	−0.19	0.31	−0.09	0.29	0.04	0.33

IPC, Interpersonal Circumplex; WRAT-3, Wide Range Achievement Test – 3; SC, Social Cognition. All continuous variables were grand-mean centered; all categorical variables were effect coded (Diagnosis is coded with NA as the reference group; race is effect coded with white as the reference group). The unstandardized regression coefficients and standard errors come from the corresponding full model in which all of the effects were included.

* p < 0.05,

** *p < 0.01*.

**Table 8:** Actor-partner interdependence model analyses estimating the combinatorial effects of diagnostic status and social skills variables on the social evaluation outcomes of closeness, interaction quality, warmth, and dominance.

	**Social evaluation outcomes**							
**Predictors**	**Closeness**		**Interaction quality**		**IPC warmth**		**IPC dominance**	
**Social skills predictors**	**b**	**SE**	**b**	**SE**	**b**	**SE**	**b**	**SE**
Intercept	2.90	0.24	5.86	0.21	−0.04	0.20	−0.23	0.22
**Demographic variables**								
Actor WRAT	−0.02	0.01	−0.01	0.01	0.00	0.01	−0.01	0.01
Actor race – AfricanAmerican	0.15	0.39	0.21	0.34	0.23	0.33	−0.28	0.36
Actor race – Asian	0.01	0.48	−0.49	0.42	−0.09	0.40	−0.05	0.44
Actor race – Other	0.05	0.33	0.53	0.29	0.04	0.28	0.42	0.32
Actor age	0.06^*^	0.03	0.04	0.03	0.06^*^	0.02	0.00	0.03
**Diagnosis variables**								
Actor diagnosis	0.31	0.16	−0.10	0.15	−0.27	0.14	0.06	0.17
Partner diagnosis	−0.02	0.16	0.09	0.15	−0.00	0.14	−0.09	0.16
Actor^*^Partner diagnosis	0.12	0.21	−0.00	0.19	0.12	0.18	0.19	0.20
**Social skills variables**								
Actor SS	−0.06	0.19	−0.18	0.17	−0.21	0.16	0.20	0.18
Partner SS	0.28	0.19	0.38^*^	0.17	0.38^*^	0.16	0.29	0.19
Actor^*^Partner SS	−0.34	0.23	−0.29	0.20	0.15	0.19	0.17	0.21
**Moderation of social skills variables by diagnosis variables**								
Actor diagnosis^*^Actor SS	0.08	0.22	0.17	0.19	0.02	0.18	−0.18	0.20
Actor diagnosis^*^Partner SS	−0.25	0.21	−0.22	0.19	0.07	0.18	−0.13	0.20
Actor diagnosis^*^(Actor^*^Partner SS)	0.40^*^	0.19	0.16	0.18	0.12	0.17	0.10	0.19
Partner diagnosis^*^Actor SS	−0.09	0.21	−0.13	0.18	0.17	0.17	0.42^*^	0.19
Partner diagnosis^*^Partner SS	−0.24	0.22	0.03	0.20	−0.27	0.19	−0.23	0.21
Partner diagnosis^*^(Actor^*^Partner SS)	0.18	0.19	0.00	0.18	−0.12	0.17	0.00	0.19
**Moderation of social skills variables by dyad type**								
(Actor^*^Partner diagnosis)^*^Actor SS	0.14	0.19	0.02	0.17	−0.03	0.16	−0.29	0.18
(Actor^*^Partner diagnosis)^*^Partner SS	0.34	0.19	−0.06	0.17	−0.13	0.16	0.02	0.19
(Actor^*^Partner diagnosis)^*^(Actor^*^Partner SS)	0.01	0.24	0.23	0.21	−0.06	0.20	−0.07	0.22

IPC, Interpersonal Circumplex; WRAT-3, Wide Range Achievement Test – 3; SS, Social Skills. All continuous variables were grand-mean centered; all categorical variables were effect coded (Diagnosis is coded with NA as the reference group; race is effect coded with white as the reference group). The unstandardized regression coefficients and standard errors come from the corresponding full model in which all of the effects were included.

* *p <0.05*.

**Table 9:** Actor-partner interdependence model analyses estimating the combinatorial effects of diagnostic status and social motivation variables on the social evaluation outcomes of closeness, interaction quality, warmth, and dominance.

	**Social evaluation outcomes**							
**Predictors**	**Closeness**		**Interaction quality**		**IPC warmth**		**IPC dominance**	
**Social motivation predictors**	***b***	**SE**	***b***	**SE**	***b***	**SE**	***b***	**SE**
Intercept	2.87	0.24	5.78	0.21	0.11	0.16	0.04	0.22
**Demographic variables**								
Actor WRAT	−0.03^*^	0.01	−0.01	0.01	0.01	0.01	−0.02^*^	0.01
Actor race – AfricanAmerican	0.32	0.40	0.08	0.34	0.28	0.27	−0.04	0.36
Actor race – Asian	0.05	0.45	−0.43	0.39	−0.21	0.30	−0.23	0.40
Actor race – Other	−0.18	0.33	0.67^*^	0.30	0.17	0.25	0.39	0.32
Actor age	0.05^*^	0.03	0.04	0.02	0.05^*^	0.02	−0.00	0.03
**Diagnosis variables**								
Actor diagnosis	0.35^**^	0.12	0.16	0.11	−0.06	0.10	−0.00	0.13
Partner diagnosis	−0.12	0.12	−0.16	0.11	−0.16	0.10	−0.09	0.12
Actor^*^Partner diagnosis	0.23	0.14	0.13	0.12	0.10	0.10	−0.03	0.13
**Social motivation variables**								
Actor SM	0.02	0.02	0.03	0.02	0.02	0.02	−0.00	0.02
Partner SM	0.01	0.02	−0.02	0.02	−0.00	0.02	−0.02	0.02
Actor^*^Partner SM	0.01	0.00	0.00	0.00	−0.00	0.00	0.00	0.00
**Moderation of social motivation variables by diagnosis variables**								
Actor diagnosis^*^Actor SM	−0.01	0.02	−0.02	0.02	0.00	0.02	0.01	0.02
Actor diagnosis^*^Partner SM	−0.00	0.02	0.01	0.02	0.00	0.02	−0.01	0.02
Actor diagnosis^*^(Actor^*^Partner SM)	−0.00	0.00	0.00	0.00	0.00	0.00	−0.00	0.00
Partner diagnosis^*^Actor SM	0.02	0.02	0.00	0.02	0.04^*^	0.02	−0.01	0.02
Partner diagnosis^*^Partner SM	0.02	0.02	0.00	0.02	−0.02	0.02	0.00	0.02
Partner diagnosis ^*^(Actor^*^Partner SM)	0.00	0.00	0.00	0.00	0.00	0.00	0.01	0.00
**Moderation of social motivation variables by dyad type**								
(Actor^*^Partner diagnosis)^*^Actor SM	−0.02	0.02	−0.00	0.02	0.02	0.02	0.00	0.02
(Actor^*^Partner diagnosis)^*^ Partner SM	−0.00	0.02	0.01	0.02	−0.01	0.02	0.04	0.02
(Actor^*^Partner diagnosis)^*^ (Actor^*^Partner SM)	−0.00	0.00	−0.00	0.00	−0.00	0.00	−0.00	0.00

IPC, Interpersonal Circumplex; WRAT-3, Wide Range Achievement Test – 3; SM, Social Motivation; All continuous variables were grand-mean centered; all categorical variables were effect coded (Diagnosis is coded with NA as the reference group; race is effect coded with white as the reference group). The unstandardized regression coefficients and standard errors come from the corresponding full model in which all of the effects were included.

* p < 0.05,

** *p <0.01*.

**Table 10:** Actor-partner interdependence model analyses estimating the combinatorial effects of diagnostic status and social cognition variables on the first impression variables of behavioral intent, awkwardness (reversed), attractiveness, and trustworthiness.

	**First-impression outcome variables**							
**Predictors**	**Behavioral intent**		**Awkwardness (reverse scored)**		**Attractiveness**		**Trustworthiness**	
**Social-cognition predictors**	***b***	**SE**	***b***	**SE**	***b***	**SE**	***b***	**SE**
Intercept	3.09	0.09	3.03	0.12	2.51	0.16	3.42	0.09
**Demographic variables**								
Actor WRAT	0.00	0.00	−0.01	0.01	0.01	0.01	0.00	0.01
Actor race – AfricanAmerican	0.13	0.17	0.32	0.22	0.11	0.30	0.04	0.17
Actor race – Asian	−0.36	0.18	−0.24	0.24	−0.04	0.32	0.15	0.18
Actor race – Other	0.27	0.14	0.03	0.21	−0.00	0.24	0.05	0.15
Actor age	0.03^*^	0.01	0.02	0.02	0.03	0.02	−0.00	0.01
**Diagnosis variables**								
Actor diagnosis	−0.04	0.06	0.06	0.09	0.08	0.11	−0.08	0.06
Partner diagnosis	−0.03	0.06	−0.28^**^	0.09	−0.16	0.10	−0.07	0.06
Actor^*^Partner diagnosis	0.14^*^	0.07	−0.03	0.09	−0.08	0.12	0.06	0.07
**Social cognition variables**								
Actor SC	0.08	0.11	0.15	0.16	0.32	0.19	−0.16	0.11
Partner SC	0.02	0.11	−0.18	0.16	0.02	0.19	−0.06	0.11
Actor^*^Partner SC	0.15	0.16	−0.01	0.21	0.08	0.28	0.06	0.16
**Moderation of social cognition variables by diagnosis variables**								
Actor diagnosis^*^Actor SC	−0.09	0.11	−0.32	0.16	−0.25	0.19	0.08	0.11
Actor diagnosis^*^Partner SC	0.11	0.11	−0.47^**^	0.17	−0.13	0.20	0.13	0.12
Actor diagnosis^*^(Actor^*^Partner SC)	−0.10	0.14	−0.03	0.21	−0.01	0.23	−0.18	0.14
Partner diagnosis^*^Actor SC	0.17	0.12	0.29	0.17	0.12	0.20	0.06	0.12
Partner diagnosis^*^Partner SC	−0.03	0.11	0.15	0.16	−0.03	0.19	−0.14	0.11
Partner diagnosis^*^(Actor^*^Partner SC)	0.28^*^	0.14	0.50^*^	0.21	0.05	0.23	0.08	0.14
**Moderation of social cognition variables by dyad type**								
(Actor^*^Partner diagnosis)^*^Actor SC	−0.09	0.12	−0.10	0.17	−0.26	0.20	−0.00	0.12
(Actor^*^Partner diagnosis)^*^Partner SC	−0.02	0.12	0.46^**^	0.17	0.08	0.20	−0.09	0.12
(Actor^*^Partner diagnosis)^*^(Actor^*^Partner SC)	−0.07	0.15	0.14	0.20	−0.34	0.27	−0.06	0.15

WRAT−3, Wide Range Achievement Test – 3; SC, Social Cognition. All continuous variables were grand-mean centered; all categorical variables were effect coded (Diagnosis is coded with NA as the reference group; race is effect coded with white as the reference group). The unstandardized regression coefficients and standard errors come from the corresponding full model in which all of the effects were included.

* p < 0.05,

** *p < 0.01*.

**Table 11:** Actor-partner interdependence model analyses estimating the combinatorial effects of diagnostic status and social skills variables on the first impression variables of behavioral intent, awkwardness (reversed), attractiveness, and trustworthiness.

	**First-impression outcome variables**							
**Predictors**	**Behavioral intent**		**Awkwardness (reverse scored)**		**Attractiveness**		**Trustworthiness**	
**Social skills predictors**	***b***	**SE**	***b***	**SE**	***b***	**SE**	***b***	**SE**
Intercept	3.09	0.10	2.98	0.13	2.60	0.17	3.48	0.10
**Demographic variables**								
Actor WRAT	0.00	0.01	−0.01	0.01	0.01	0.01	0.01	0.01
Actor race – AfricanAmerican	0.20	0.16	−0.06	0.22	0.05	0.28	0.19	0.17
Actor race – Asian	−0.30	0.20	−0.21	0.27	−0.27	0.34	0.13	0.20
Actor race – Other	0.15	0.14	0.25	0.20	0.21	0.23	−0.12	0.15
Actor age	0.02	0.01	0.01	0.02	0.02	0.02	−0.01	0.01
**Diagnosis variables**								
Actor diagnosis	0.05	0.08	−0.08	0.13	−0.03	0.12	0.10	0.08
Partner diagnosis	−0.06	0.07	−0.18	0.12	−0.04	0.12	−0.14	0.08
Actor*Partner diagnosis	0.09	0.09	0.06	0.12	−0.25	0.15	−0.09	0.09
**Social skills variables**								
Actor SS	0.03	0.08	−0.08	0.13	0.00	0.13	0.04	0.09
Partner SS	0.01	0.08	0.38^**^	0.13	0.21	0.13	−0.07	0.09
Actor*Partner SS	−0.17	0.09	−0.04	0.13	−0.17	0.16	−0.07	0.10
**Moderation of social skills variables by diagnosis variables**								
Actor diagnosis*Actor SS	−0.02	0.09	−0.04	0.13	0.11	0.15	0.08	0.10
Actor diagnosis*Partner SS	−0.07	0.09	0.02	0.13	−0.29	0.15	−0.10	0.10
Actor diagnosis*(Actor*Partner SS)	−0.03	0.09	0.09	0.14	0.26	0.14	−0.08	0.09
Partner diagnosis*Actor SS	−0.02	0.09	0.03	0.13	−0.23	0.15	−0.04	0.09
Partner diagnosis*Partner SS	0.01	0.09	−0.09	0.14	0.22	0.16	0.11	0.10
Partner diagnosis *(Actor*Partner SS)	0.05	0.09	−0.00	0.14	−0.06	0.14	0.06	0.09
**Moderation of social skills variables by dyad type**								
(Actor*Partner diagnosis)*Actor SS	−0.01	0.08	−0.00	0.13	0.17	0.13	−0.10	0.09
(Actor*Partner diagnosis)*Partner SS	0.06	0.08	−0.03	0.13	−0.00	0.13	0.11	0.09
(Actor*Partner diagnosis)* (Actor*Partner SS)	0.05	0.10	−0.07	0.13	0.23	0.17	0.02	0.10

WRAT-3, Wide Range Achievement Test – 3; SS, Social Skills. All continuous variables were grand-mean centered; all categorical variables were effect coded (Diagnosis is coded with NA as the reference group; race is effect coded with white as the reference group). The unstandardized regression coefficients and standard errors come from the corresponding full model in which all of the effects were included.

** *p < 0.01*.

**Table 12:** Actor-partner interdependence model analyses estimating the combinatorial effects of diagnostic status and social motivation variables on the first impression variables of behavioral intent, awkwardness (reversed), attractiveness, and trustworthiness.

	**First-impression outcome variables**							
**Predictors**	**Behavioral intent**		**Awkwardness (reverse scored)**		**Attractiveness**		**Trustworthiness**	
**Social Motivation Predictors**	***b***	**SE**	***b***	**SE**	***b***	**SE**	***b***	**SE**
Intercept	3.09	0.09	3.14	0.14	2.55	0.16	3.48	0.09
**Demographic variables**								
Actor WRAT	0.00	0.00	−0.01	0.01	0.01	0.01	0.01	0.00
Actor race – AfricanAmerican	0.17	0.15	−0.00	0.23	−0.11	0.26	0.03	0.15
Actor race – Asian	−0.31	0.16	−0.15	0.26	0.08	0.30	0.30	0.16
Actor race – Other	0.20	0.13	0.22	0.21	0.20	0.23	−0.04	0.14
Actor age	0.02	0.01	0.02	0.02	0.02	0.02	−0.00	0.01
**Diagnosis variables**								
Actor diagnosis	0.06	0.05	0.10	0.09	0.07	0.09	−0.02	0.06
Partner diagnosis	−0.03	0.05	−0.43^**^	0.09	−0.20^*^	0.08	0.02	0.05
Actor^*^Partner diagnosis	0.13^*^	0.05	−0.01	0.08	−0.03	0.09	−0.03	0.05
**Social motivation variables**								
Actor SM	0.02^*^	0.01	0.01	0.02	0.01	0.01	−0.01	0.01
Partner SM	0.00	0.01	−0.03	0.02	−0.01	0.01	0.01	0.01
Actor^*^Partner SM	0.00	0.00	−0.00	0.00	−0.01^*^	0.00	−0.00^*^	0.00
**Moderation of social motivation variables by diagnosis variables**								
Actor diagnosis^*^Actor SM	−0.00	0.01	−0.00	0.01	−0.03	0.02	0.01	0.01
Actor diagnosis^*^Partner SM	0.01	0.01	−0.01	0.01	−0.02	0.02	0.00	0.01
Actor diagnosis^*^(Actor^*^Partner SM)	0.00	0.00	−0.00	0.00	−0.00	0.00	0.00	0.00
Partner diagnosis^*^Actor SM	0.00	0.01	0.01	0.01	−0.01	0.02	−0.01	0.01
Partner diagnosis^*^Partner SM	−0.00	0.01	0.01	0.01	−0.01	0.02	−0.01	0.01
Partner diagnosis^*^(Actor^*^Partner SM)	0.00	0.00	0.00	0.00	0.00	0.00	0.00	0.00
**Moderation of social motivation variables by dyad type**								
(Actor^*^Partner diagnosis)^*^Actor SM	0.01	0.01	−0.00	0.02	0.00	0.01	0.03^**^	0.01
(Actor^*^Partner diagnosis)^*^Partner SM	−0.00	0.01	0.02	0.01	0.02	0.01	−0.00	0.01
(Actor^*^Partner diagnosis)^*^ (Actor^*^Partner SM)	−0.00	0.00	0.00	0.00	0.00	0.00	0.00	0.00

WRAT-3, Wide Range Achievement Test – 3; SM, Social Motivation. All continuous variables were grand-mean centered; all categorical variables were effect coded (Diagnosis is coded with NA as the reference group; race is effect coded with white as the reference group). The unstandardized regression coefficients and standard errors come from the corresponding full model in which all of the effects were included.

* p < 0.05,

** *p < 0.01*.

**Table 13:** Actor-partner interdependence model analyses estimating the combinatorial effects of diagnostic status and social cognition variables on the first impression variables of aggressiveness/dominance, smartness, and liking.

	**First-impression outcome variables**					
**Predictors**	**Aggressiveness/Dominance**		**Smart**		**Liking**	
**Social-cognition predictors**	***b***	**SE**	***b***	**SE**	***b***	**SE**
Intercept	1.74	0.10	3.46	0.14	3.35	0.09
**Demographic variables**						
Actor WRAT	−0.00	0.01	−0.01	0.01	0.00	0.01
Actor race – AfricanAmerican	0.05	0.18	−0.30	0.25	0.16	0.15
Actor race – Asian	0.08	0.20	0.25	0.27	−0.37^*^	0.17
Actor race – Other	0.05	0.18	0.26	0.22	0.30^*^	0.15
Actor age	0.01	0.02	0.06^**^	0.02	0.02	0.01
**Diagnosis variables**						
Actor diagnosis	0.16	0.09	−0.14	0.09	−0.06	0.07
Partner diagnosis	0.06	0.08	−0.04	0.09	0.01	0.07
Actor^*^Partner diagnosis	−0.00	0.07	0.21^*^	0.10	0.01	0.06
**Social cognition variables**						
Actor SC	0.13	0.15	0.05	0.17	0.08	0.12
Partner SC	0.07	0.15	−0.02	0.17	0.02	0.12
Actor^*^Partner SC	0.01	0.17	−0.00	0.23	−0.01	0.15
**Moderation of social cognition variables by diagnosis variables**						
Actor diagnosis^*^Actor SC	−0.13	0.15	0.06	0.16	−0.17	0.12
Actor diagnosis^*^Partner SC	−0.08	0.15	0.07	0.17	0.04	0.13
Actor diagnosis ^*^(Actor^*^Partner SC)	0.10	0.20	−0.29	0.21	−0.03	0.17
Partner diagnosis^*^Actor SC	−0.01	0.16	0.26	0.18	0.18	0.13
Partner diagnosis^*^Partner SC	−0.18	0.15	0.25	0.16	0.01	0.12
Partner diagnosis^*^(Actor^*^Partner SC)	−0.02	0.20	0.20	0.21	0.33	0.17
**Moderation of social cognition variables by dyad type**						
(Actor^*^Partner diagnosis)^*^ Actor SC	0.06	0.16	−0.25	0.18	−0.08	0.13
(Actor^*^Partner diagnosis)^*^Partner SC	0.10	0.15	−0.01	0.17	0.12	0.13
(Actor^*^Partner diagnosis)^*^(Actor^*^Partner SC)	−0.44^*^	0.17	0.14	0.23	−0.18	0.14

WRAT−3, Wide Range Achievement Test – 3; SC, Social Cognition. All continuous variables were grand–mean centered; all categorical variables were effect coded (Diagnosis is coded with NA as the reference group; race is effect coded with white as the reference group). The unstandardized regression coefficients and standard errors come from the corresponding full model in which all of the effects were included.

* p < 0.05,

** *p <0.01*.

**Table 14:** Actor-partner interdependence model analyses estimating the combinatorial effects of diagnostic status and social skills variables on the first impression variables of aggressiveness/dominance, smartness, and liking.

	**First-impression outcome variables**					
**Predictors**	**Aggressiveness/Dominance**		**Smart**		**Liking**	
**Social skills predictors**	***b***	**SE**	***b***	**SE**	***b***	**SE**
Intercept	1.79	0.11	3.30	0.15	3.35	0.10
**Demographic variables**						
Actor WRAT	−0.01	0.01	−0.00	0.01	0.00	0.01
Actor race – AfricanAmerican	0.00	0.18	−0.22	0.24	0.13	0.17
Actor race – Asian	−0.02	0.22	0.28	0.30	−0.35	0.21
Actor race – Other	0.04	0.17	0.28	0.21	0.28	0.16
Actor age	0.02	0.02	0.03	0.02	0.02	0.01
**Diagnosis variables**						
Actor diagnosis	0.01	0.12	0.13	0.11	−0.04	0.10
Partner diagnosis	0.07	0.12	−0.13	0.11	−0.01	0.09
Actor^*^Partner diagnosis	−0.02	0.10	0.21	0.13	−0.06	0.09
**Social skills variables**						
Actor SS	−0.07	0.12	0.24	0.12	0.01	0.10
Partner SS	−0.01	0.12	−0.07	0.12	0.00	0.10
Actor^*^Partner SS	0.14	0.10	−0.08	0.14	0.02	0.10
**Moderation of social skills variables by diagnosis variables**						
Actor diagnosis^*^Actor SS	0.18	0.12	−0.22	0.14	0.05	0.10
Actor diagnosis^*^Partner SS	−0.04	0.12	0.12	0.14	0.08	0.10
Actor diagnosis^*^(Actor^*^Partner SS)	−0.01	0.13	−0.16	0.13	−0.08	0.11
Partner diagnosis^*^Actor SS	−0.06	0.11	0.07	0.13	−0.10	0.10
Partner diagnosis^*^Partner SS	0.03	0.12	0.02	0.14	−0.02	0.11
Partner diagnosis^*^(Actor^*^Partner SS)	−0.01	0.13	0.21	0.13	0.06	0.11
**Moderation of social skills variables by dyad type**						
(Actor^*^Partner diagnosis)^*^ Actor SS	0.08	0.12	−0.14	0.12	−0.04	0.10
(Actor^*^Partner diagnosis)^*^Partner SS	−0.00	0.12	0.10	0.12	0.02	0.10
(Actor^*^Partner diagnosis)^*^ (Actor^*^Partner SS)	−0.04	0.11	0.11	0.14	0.03	0.10

*WRAT-3, Wide Range Achievement Test – 3; SS, Social Skills. All continuous variables were grand-mean centered; all categorical variables were effect coded (Diagnosis is coded with NA as the reference group; race is effect coded with white as the reference group). The unstandardized regression coefficients and standard errors come from the corresponding full model in which all of the effects were included*.

**Table 15:** Actor-partner interdependence model analyses estimating the combinatorial effects of diagnostic status and social motivation variables on the first impression variables of aggressiveness/dominance, smartness, and liking.

	**First-impression outcome variables**					
**Predictors**	**Aggressiveness/Dominance**		**Smart**		**Liking**	
**Social motivation predictors**	***b***	**SE**	***b***	**SE**	***b***	**SE**
Intercept	1.78	0.12	3.43	0.14	3.34	0.09
**Demographic variables**						
Actor WRAT	−0.01	0.01	−0.01	0.01	0.01	0.01
Actor race – AfricanAmerican	−0.07	0.19	−0.12	0.24	−0.01	0.14
Actor race – Asian	0.18	0.21	0.22	0.27	−0.26	0.15
Actor race – Other	0.04	0.18	0.19	0.21	0.36^*^	0.14
Actor age	0.01	0.02	0.04^*^	0.02	0.02	0.01
**Diagnosis variables**						
Actor diagnosis	0.00	0.08	0.03	0.08	−0.02	0.07
Partner diagnosis	0.05	0.08	0.00	0.08	−0.02	0.06
Actor^*^Partner diagnosis	0.08	0.07	0.09	0.09	−0.00	0.05
**Social motivation variables**						
Actor SM	−0.02	0.01	0.02	0.01	0.01	0.01
Partner SM	0.01	0.01	0.01	0.01	−0.01	0.01
Actor^*^Partner SM	−0.00	0.00	−0.00	0.00	−0.00^*^	0.00
**Moderation of social motivation variables by diagnosis variables**						
Actor diagnosis^*^Actor SM	−0.00	0.01	0.01	0.01	−0.02	0.01
Actor diagnosis^*^Partner SM	−0.00	0.01	0.01	0.01	0.01	0.01
Actor diagnosis^*^(Actor^*^Partner SM)	0.00	0.00	−0.00	0.00	0.00	0.00
Partner diagnosis^*^Actor SM	−0.00	0.01	−0.01	0.01	0.01	0.01
Partner diagnosis^*^Partner SM	−0.00	0.01	−0.01	0.01	−0.01	0.01
Partner diagnosis^*^ (Actor^*^Partner SM)	−0.00	0.00	0.00	0.00	0.00	0.00
**Moderation of social motivation variables by dyad type**						
(Actor^*^Partner diagnosis)^*^Actor SM	0.00	0.01	0.00	0.01	0.01	0.01
(Actor^*^Partner diagnosis)^*^Partner SM	0.00	0.01	0.01	0.01	0.00	0.01
(Actor^*^Partner diagnosis)^*^(Actor^*^Partner SM)	0.00	0.00	−0.00	0.00	0.00	0.00

WRAT−3, Wide Range Achievement Test – 3; SM, Social Motivation. All continuous variables were grand-mean centered; all categorical variables were effect coded (Diagnosis is coded with NA as the reference group; race is effect coded with white as the reference group). The unstandardized regression coefficients and standard errors come from the corresponding full model in which all of the effects were included.

* *p < 0.05*.

For ease of presentation, we grouped the regression coefficients and standard errors for each set of predictors into different sections: (1) demographic variables, (2) diagnosis variables (i.e., the actor, partner, and actor-partner interaction effects for diagnostic status), (3) social ability variables (i.e., the actor, partner, and actor-partner interactions effects for the social ability variables), (4) moderation of the social ability variables by diagnosis variables (i.e., whether the actor, partner, and actor-partner interactions effects for the social ability variables depend upon the participants' or their partners' diagnostic status), and (5) moderation of the social ability variables by diagnostic combination or dyad type (i.e., whether the actor, partner, and actor-partner interactions effects for the social ability variables depend upon the diagnostic composition of the dyad). The regression coefficients and standard errors reflect the estimates from models wherein all predictors are included. Given the presence of multiple interaction terms, tolerance values (indices for multicollinearity) were rather low for the terms involving the social ability variables (mean values ranged from 0.26 for the social skill variables to 0.41 for the social motivation variables), moderation of the social ability variables by the diagnosis variables (mean values ranged from 0.25 for the social skill variables to 0.43 for the social motivation variables), and moderation of the social ability variables by diagnostic combination (mean values ranged from 0.26 for the social skill variables to 0.41 for the social motivation variables). Given our focus on the interaction terms, these tolerance values do not pose a problem (see McClelland et al., 2017).

#### Effects of Social Cognition on Interaction Outcomes

There were no significant actor, partner, or actor-partner interaction effects of social-cognition on any of the outcomes (see Tables 7, 10, 13). Nevertheless, we found a significant three-way interaction between actor diagnosis, partner diagnosis, and actor social cognition on interaction quality. To break this down, we inspected the simple two-way interactions between partner diagnosis and actor social cognition for autistic and NA participants. These analyses revealed an effect for NA actors (*b* = 1.13, SE = 0.43, *p* = 0.01) but not autistic actors (*b* = −0.26, SE = 0.19, *p* = 0.18). Within A-NA dyads, NA actors with higher social cognitive performance rated the interaction quality higher (*b* = 1.80, SE = 0.72, *p* = 0.014). However, this pattern was not observed for NA actors within NA-NA dyads (*b* = −0.45, SE = 0.43, *p* = 0.30).

We also found a significant two-way interaction of actor diagnosis and partner social cognition on awkwardness scores. Autistic adults rated partners with higher social cognitive performance as more awkward than partners with lower social cognitive performance (*b* = −0.65, SE = 0.27, *p* = 0.02). This pattern was not observed for NA adults (*b* = 0.29, SE = 0.19, *p* = 0.13). However, this interaction was subsumed by a three-way interaction of actor and partner diagnosis and partner social cognitive ability. To break this down, we first inspected the simple two-way interactions of partner diagnosis and partner social cognition for autistic actors and for NA actors. The two-way interaction was significant for autistic actors (*b* = 0.61, SE = 0.27, *p* = 0.03) but not NA actors (*b* = −0.31, SE = 0.19, *p* = 0.10). Further breaking down the two-way interaction for autistic actors revealed an effect of partner social cognition on autistic actors' awkwardness ratings in mixed dyads but not dyads of the same diagnosis. Specifically, whereas autistic actors rated their NA partners as more awkward when their partner had higher social cognitive performance (*b* = −1.26, SE = 0.52, *p* = 0.02), this effect was not seen for autistic actors in A-A dyads (*b* = −0.03, SE = 0.15, *p* = 0.81). No other moderating effects were observed.

#### Exploratory Analyses: Effects of Individual Social Cognitive Domains on Interaction Outcomes

In addition to examining effects of the overall social cognition composite, we explored the effects of performance on each individual social cognitive task (i.e., Benton, ER40, TASIT). There were significant two-way interactions of actor and partner diagnosis with partner emotion recognition abilities (i.e., ER-40 scores) for trustworthiness ratings. Autistic actors trusted their partners more when their partners had higher levels of emotion recognition ability (*b* = 0.10, SE = 0.03, *p* = 0.003). This effect was not significant for NA actors (*b* = −0.03, SE = 0.03, *p* = 0.296). Additionally, participants rated NA participants with stronger emotion recognition abilities as more trustworthy (*b* = 0.09, SE = 0.03, *p* = 0.006), but this effect was not significant for autistic partners with differing levels of emotion recognition ability (*b* = −0.04, SE = 0.03, *p* = 0.15).

There were also significant two-way interactions for likeability. Autistic actors liked partners more when their partners had higher emotion recognition abilities (*b* = 0.09, SE = 0.03, *p* = 0.009), but this effect was not significant for NA actors (*b* = −0.03, SE = 0.03, *p* = 0.31). The interaction of partner diagnosis with facial recognition scores (i.e., Benton) was also significant (*p* = 0.002). Following up this interaction with simple slopes revealed that the effect of Benton scores on likeability ratings did not significantly differ from zero for both autistic and NA partners, but the pattern of effects suggests that participants rated higher likeability for NA partners who had lower facial recognition scores (NA partner: *b* = −0.04, SE = 0.02, *p* = 0.08) and for autistic adults who had higher facial recognition scores (A partner: *b* = 0.03, SE = 0.02, *p* = 0.08).

#### Effects of Social Skills on Interaction Outcomes

There was a significant effect of the partner's composite social skills rating on awkwardness evaluations (*p* < 0.001), such that partners who were higher on observed social skills were rated as less awkward. No other actor, partner, or actor-partner interaction effects involving social skills were significant, and there was no evidence that diagnostic status or dyad type moderated any of these effects (see Tables 8, 11, 14).

#### Effects of Social Motivation on Interaction Outcomes

There were no significant actor, partner, or actor-partner interactions for the social motivation variables on any of the social interaction outcome variables (see Tables 9, 12, 15). However, there was a significant three-way interaction of actor and partner diagnoses with actor motivation scores on trustworthiness ratings (*p* = 0.007). To break this down, we examined the simple two-way interactions between partner diagnosis and actor social motivation for autistic and NA actors. There was a significant interaction of partner diagnosis with actor social motivation for NA actors (*b* = −0.03, SE = 0.02, *p* = 0.03). NA actors with more social motivation rated their autistic partners as less trustworthy (*b* = −0.06, SE = 0.03, *p* = 0.04), but this did not extend to NA partners (*b* = 0.01, SE = 0.01, *p* = 0.45). Moreover, the interaction of partner diagnosis and social motivation was marginally significant for autistic actors (*b* = 0.02, SE = 0.01, *p* = 0.06). Breaking this two-way interaction down further revealed that autistic actors with more social motivation rated other autistic adults as marginally more trustworthy (*b* = 0.02, SE = 0.01, *p* = 0.06), but this effect did not extend to NA partners (*b* = −0.02, SE = 0.02, *p* = 0.26).

## Discussion

In a previous study using this sample (Morrison et al., 2020), autistic adults were evaluated less favorably by unfamiliar partners following a “get to know you” conversation, and NA participants were less interested than autistic participants in interacting with them again in the future. In the current study, these autistic adults performed lower on a composite of social cognitive measures, were rated as less normative on social skills, and endorsed fewer normative indicators of social motivation compared to NA adults. All of these findings align with previous research (Chevallier et al., 2012a; Morrison et al., 2017, 2019a; Sasson et al., 2017; DeBrabander et al., 2019), but contrary to expectation, only minimal links were found between autistic adults' performance on the three social ability domains and their social interaction outcomes. In some cases, it was the social abilities of NA adults, not those of autistic adults, that were most predictive of outcomes, and this was particularly the case when they were interacting with autistic people. NA social cognition, for instance, predicted some of their interaction outcomes (e.g., awkwardness, interaction quality) with autistic but not NA partners. Collectively, findings suggest that standalone measures of autistic social abilities are not particularly predictive of their poorer interaction outcomes with NA partners. Rather, more consistent with relational accounts of autistic sociability (Milton, 2012; Bottema-Beutel, 2017; Bolis et al., 2018; Redcay and Schilbach, 2019), the dyadic combination of social abilities between diagnostic groups was more predictive of how autistic and NA adults evaluated (and were evaluated by) their partners.

Across the three social abilities assessed here, only normative social skill demonstrated any unidirectional predictive value on interaction outcomes. Most notably, those who were coded as less normative in their overall social skill were evaluated as more awkward. It may be the case that the overall social skill rating used here (Pinkham and Penn, 2006) is driven in part by the coder's perception of the person's awkwardness, which tended to align with participant evaluations of awkwardness within the dyads. This interpretation may explain why the social skill measure was associated with awkwardness ratings but not other evaluated traits: “awkwardness” may be consistent with an individual's judgment of another person's social skill, with lower ratings signifying a deviation from normative social expression and behavior. Perhaps not coincidently, NA raters in previous studies have tended to discriminate autistic and NA participants more on awkwardness than any other trait judgment (Grossman, 2015; Sasson et al., 2017; Sasson and Morrison, 2019), with awkwardness ratings highly associated with a reluctance among NA adults to pursue subsequent social interaction.

Autistic raters in this study also judged autistic people high on awkwardness, but unlike NA raters, this judgment was not associated with reduced social interest (Morrison et al., 2020). What underlies this dissociation remains unclear. Future research may seek to isolate the specific characteristics and cues driving higher scores of awkwardness and assess whether they may be interpreted and valued differently by autistic and NA people. For instance, recent findings suggest that autistic people may seek out interaction with those who present and communicate atypically (Granieri et al., 2020), as these differences—ones often described as “awkward” —may cohere with their social preferences and facilitate better interpersonal communication and connection. Similarly, Heasman and Gillespie (2019) found that, contrary to normative expectations, misunderstanding, and misinterpretation among autistic adults did not invariably lead to deterioration of the interaction. Viewed through a conventional social lens, such disruptions may be perceived as awkward or seen as evidence of social disjunction, but these instances may be experienced differently by autistic adults. Perceptions of “awkwardness” therefore may reflect just one of many differences in social expectations and experiences between autistic and NA people.

Aside from ratings of awkwardness, normative social skill did not predict any trait evaluations or interaction outcomes for either the individual or their partner, was no more predictive of outcomes for autistic compared to NA adults, and did not vary across different dyad combinations. It may be the case that broader social judgments within a “get to know you” conversation depend less upon observable social skill and more upon other characteristics and considerations. For example, ratings of traits such as attractiveness may be influenced more by physical attributes rather than social behaviors, and judgments of likeability, trustworthiness, warmth, and interaction quality may be more related to conversational content, personal disclosure, and interpersonal alignment. Alternatively, or perhaps complementarily, conceptualizing social skill as an objective metric in which individuals can be quantitatively rank ordered and a single standard applied to all populations may be unhelpful for predicting complex social relationship dynamics, particularly between neurologically diverse people (Heerey, 2015; Bottema-Beutel, 2017; Milton, 2017). What constitutes good “social skill” may vary across groups and individuals, and a single holistic social skill rating may simply be inadequate for capturing and summarizing social skill across an entire dynamic and emergent interaction. Indeed, work examining interpersonal warmth and dominance suggests that moment to moment behaviors rather than overall summaries are better predictors of interaction outcomes (Markey et al., 2010; Stevanovic et al., 2017).

Autistic adults' social cognitive performance was also not particularly predictive of their interaction outcomes. In fact, within mixed dyads, it was the social cognitive performance of NA adults, not autistic adults, that generated most of the effects. For instance, better social cognitive performance among NA adults was associated with rating conversations with autistic partners as higher in quality. One possible interpretation is that social cognitive ability among NA adults may facilitate better perception of social cues from their autistic partners and mitigate the difficulties NA people often have inferring autistic mental states (Edey et al., 2016; Sheppard et al., 2016; Gernsbacher et al., 2017). This also suggests that interaction quality between autistic and NA adults may improve by increasing social cognitive ability among NA people—perhaps more so than among autistic people given that no corresponding effect was found for autistic participants. Indeed, some emerging evidence indicates that NA observers who are better able to infer autistic mental states (Alkhaldi et al., 2019) and have greater understanding about autism (Sasson and Morrison, 2019) evaluate autistic people more favorably, suggesting that social experiences of autistic people within NA environments may improve with greater NA understanding about autism. Less provocatively, higher social cognitive performance among NA adults in this study may have been a proxy for other characteristics associated with more enjoyable conversational experiences with autistic partners, like higher social engagement, attentiveness, and desire for connection. Regardless of interpretation, however, this finding of NA social cognition predicting outcomes with autistic partners was not hypothesized and should therefore be interpreted cautiously until replicated.

Although NA adults with higher social cognitive performance rated conversations with autistic partners as higher in quality, autistic participants did not share this assessment and instead actually perceived NA adults who scored better on social cognitive measures as more awkward than those who scored lower. At first blush, this finding appears counterintuitive and potentially spurious, but the strict alpha level reduces the likelihood that this is the case. What underlies this effect is unclear, but it may be the case that social cognitive ability among NA individuals manifests in social behaviors perceived as awkward or intrusive by autistic adults. Alternatively, as suggested previously, autistic individuals may interpret the term “awkward” differently than NA individuals, but other findings suggest that autistic adults did perceive “awkward” as a negative characteristic—their ratings of awkwardness were related to lower intentions to interact as well as with other less favorable trait evaluations. Importantly, however, these relationships were weaker than those found for NA adults.

Additionally, despite performing lower on several social cognitive tasks, autistic adults largely mirrored NA adults in forming less favorable evaluations of other autistic adults (Morrison et al., 2020). Thus, contrary to what might be expected based on their lower social cognitive performance, autistic adults appeared just as sensitive to social presentation differences among autistic adults and interpreted these differences similarly to their NA counterparts. Additionally, autistic adults rated partners who were more skilled in emotion recognition ability as more trustworthy and likable. This suggests that despite performing less well on standalone social cognitive tasks, interacting with someone skilled in these domains improved how autistic adults perceived their partner. Taken together, these findings suggest that the lower social cognitive performance demonstrated by autistic adults did not correspond in clear and predictable ways to their real-world social interaction outcomes. Isolated computerized assessments of social cognition such as those used here may not fully capture how these social abilities influence actual social interaction. This does not mean that these measures fail to capture social cognitive differences; in fact, as in previous research (Morrison et al., 2019b), they differentiated autistic and NA participants and were somewhat predictive of NA outcomes. Nevertheless, current findings raise questions about the mechanistic link between reduced social cognitive performance by autistic adults on standalone tasks and their difficulties interacting with NA adults. Recognizing faces and emotions from static images may not translate in presumed ways to the much more complex nature of dynamic interaction, even within NA-NA interactions. Similarly, higher social cognitive performance by NA adults did not facilitate better mutual interaction quality or an increase in shared positive outcomes with autistic adults. Collectively, such findings are consistent with double empathy (Milton, 2012) and dialectical misattunement (Bolis et al., 2018) theories of social disconnection between autistic and NA people and suggest that traditional conceptualizations of social cognitive ability may not extend in anticipated ways to autistic-NA interactions.

For social motivation, moderated results suggested lower social motivation scores among autistic participants did not impact how they were evaluated in the conversation. Indeed, there was only one group effect of social motivation, such that NA adults high on social motivation trusted their autistic partners less. It may be the case that socially motivated NA adults strive but struggle to connect with their autistic partners and misinterpret autistic social differences as indicative of lower trustworthiness. If so, this process could have adverse consequences for the social experiences of autistic adults, whose differences in social expressivity could be misperceived in ways that reinforce reluctance of NA adults to interact with them. Such an interpretation, however, is currently speculative and worthy of verification in future study.

Taken together, results from this study challenge traditional thinking about the mechanisms of social interaction difficulties for autistic adults. Typically, because on average autistic adults perform lower than NA controls on traditional social cognitive tasks (Morrison et al., 2019b), deviate in their social behavior and presentation from prototypical social skills (Morrison et al., 2017), and often report lower or different social motivation (Chevallier et al., 2012a), psychosocial treatments often seek to train autistic people to be more normative in these areas with the hope doing so will translate to greater social interaction success in the real-world (Bishop-Fitzpatrick et al., 2014; Kern Koegel et al., 2016). However, this result does not regularly occur in practice (Gates et al., 2017; Bottema-Beutel et al., 2018). Recent empirical work has shown that social cognitive performance and social skill among autistic adults on standardized measures demonstrate only a small correspondence to their functional outcomes beyond other factors (Sasson et al., 2020), and some autistic people can exhibit normative social skill despite lower theory of mind performance through cognitive compensation (Livingston et al., 2019). Indeed, among autistic adults without intellectual disability, general cognition is far more predictive of social skill than social cognition (Sasson et al., 2020), and performance on explicit social cognitive measures like the ones used here may be less predictive of social communication and interaction behavior in autism than implicit social cognitive performance (Keifer et al., 2020). Taken at face value, results from this study suggest that social cognition, social skill, and social motivation may not be useful treatment targets for improving autistic adults' initial social interactions with NA people. Alternatively, they may still influence real-life social outcomes in autism, but each were either poorly measured in the current study or done so in a way that has limited application to interaction outcomes. From this perspective, the field may improve from the development of more real-world assessments of social cognitive, social motivational, and social skills abilities, rather than continuing to rely solely on paper and pencil and computerized tasks.

The field may also benefit from exploring how other abilities and behaviors of autistic adults may be predictive of how they are evaluated. Some recent work has argued that much remains unknown about social interaction in autism (Bottema-Beutel, 2017; Bottema-Beutel et al., 2018) and has suggested applying new theoretical frameworks for understanding autistic social interaction (Bottema-Beutel, 2017). For example, (Bottema-Beutel, 2017) contends that research on social abilities should be examined using sociolinguistic approaches (e.g., conversation analysis) which not only takes the individual's context into account, but also allows for more dynamic assessment of how a person interacts with his or her environment. Additionally, this kind of approach allows for the examination of environmental and societal influences such as stigma that may play a role in how social disability develops and manifests, as well as determining the efficacy of current interventions for treating social disability (Bottema-Beutel et al., 2018). Moreover, given the heterogeneity of autism, a more person-centered approach may better approximate understanding of social difficulties than the group-level assessments and analyses used in this study and most prior work.

This is particularly important because the group-level dyadic analyses pursued here may have been under-powered to detect some effects. The sample size was determined based upon medium to large effects reported in prior interaction studies, but these may have been artificially inflated because of their smaller sample sizes (Usher et al., 2018) or because they examined different populations like the Broad Autism Phenotype (Faso et al., 2016). As a result, the effects here may have been smaller than the medium or large effects that were anticipated, and thus may not have been detectable with the current sample size of 55 dyads. Relatedly, null effects from this study should not be treated as definitive, as some may have reached statistical significance with increased power. Another limitation of the current study is that it used only a few of the social cognitive, social motivational, and social skills assessments that exist, and these may not have been the best measures to capture meaningful relationships within real-world interaction. Further, using social skill and social cognitive composite scores may have obscured more nuanced effects of specific subcomponent abilities. However, exploratory analyses assessing the effect of performance on individual social cognitive tasks also found few links to interaction outcomes. Further, it is possible that other individual mechanisms not assessed here, such as linguistic abilities and executive functioning, may also have been related to outcomes. Moreover, because some of the effects found in this study were relational and not individual, future studies may seek to move beyond examining individual predictors of social interaction outcomes to instead focus on relational variables, like interpersonal synchrony, compatibility, and affiliation.

Effects may also have been smaller than anticipated because of selection biases in the sample: most autistic participants were students attending college or a professional training program and therefore may have been more independent, intellectually capable, and socially skilled than other autistic adults. Nevertheless, they performed comparably to other autistic samples on measures of social cognition (Bishop-Fitzpatrick et al., 2017; Morrison et al., 2019b), normative social skill (Ratto et al., 2011; Morrison et al., 2017), and social motivation (Sedgewick et al., 2016), suggesting they were largely representative in terms of their measured social abilities. Additionally, because NA participants were mostly psychology students attending a university with a sizeable autistic population, they may have more experience with autism than the general population. Finally, because adequately examining the complicating effects of gender on social interaction outcomes would necessitate a prohibitive increase in sample size and additional dyadic conditions, this study was limited to studying interaction between males. Participants were also disproportionately white because of the racial breakdown of our autism recruitment sources. The lack of gender and ethnic diversity in our sample is perhaps the largest limitation of the current study. Gender and race are highly salient characteristics within social interactions, and their effects were not explored here. We hope that future studies can leverage more diverse populations to assess how the findings in this study may differ within all female dyads, as well as within cross-gender and cross-racial interactions. In particular, results may be expected to differ for autistic females, who often diverge from autistic males in some aspects of social motivation and behavior (Hull et al., 2017) and tend be evaluated more favorably than autistic males by NA individuals (Cage and Burton, 2019; Cola et al., 2020).

In summary, the current study represents the first comprehensive attempt to directly assess whether and how individual performance on measures of social cognition, social skill, and social motivation among autistic adults predicts their real-world social interaction outcomes with unfamiliar autistic and NA adults. Despite performing lower than NA participants on these measures, autistic adults' performance on each of the three social ability domains was largely unassociated with how autistic adults evaluated—and were evaluated by—their conversation partner. Contrary to prediction, in some cases the social abilities of NA adults were actually more predictive. Taken together, findings from this study raise questions about the predictive utility of standalone measures of social abilities in autistic people for understanding their social interaction difficulties with NA people. Future research should seek to examine and validate measures of real-world social cognition, social skill, and social motivation within an interactive context, and continue to emphasize relational rather than individual predictors of social interaction success.

## Data Availability Statement

The raw data supporting the conclusions of this article will be made available by the authors, without undue reservation.

## Ethics Statement

The studies involving human participants were reviewed and approved by The University of Texas at Dallas Institutional Review Board. The patients/participants provided their written informed consent to participate in this study.

## Author Contributions

KM led the design of the study, with contributions from RA and NS. KM gathered and organized the data, with contributions from KD and DJ. KM conducted data analysis in collaboration with RA. KM interpreted the data, with help from KD, DJ, RA, and NS. KM and NS wrote the manuscript, with contributions from RA, KD, and DJ. All authors contributed to the article and approved the submitted version.

## Conflict of Interest

The authors declare that the research was conducted in the absence of any commercial or financial relationships that could be construed as a potential conflict of interest.
